# Proof of concept: digital clock drawing behaviors prior to transcatheter aortic valve replacement may predict length of hospital stay and cost of care

**DOI:** 10.37349/emed.2021.00036

**Published:** 2021-04-30

**Authors:** Margaret Ellenora Wiggins, Catherine Dion, Erin Formanski, Anis Davoudi, Shawna Amini, Kenneth M. Heilman, Dana Penney, Randall Davis, Cynthia W. Garvan, George J. Arnaoutakis, Patrick Tighe, David J. Libon, Catherine C. Price

**Affiliations:** 1 Department of Clinical and Health Psychology, University of Florida, Gainesville, FL 32610, USA; 2 Department of Biomedical Engineering, University of Florida, Gainesville, FL 32611, USA; 3 Department of Anesthesiology, University of Florida College of Medicine, Gainesville, FL 32610, USA; 4 Department of Neurology, University of Florida College of Medicine, Gainesville, FL 32610, USA; 5 Department of Neurology, Lahey Hospital and Medical Center, Boston, Mass 02421, USA; 6 Department of Electronical Engineering and Computer Science, Massachusetts Institute of Technology, Cambridge, Mass 02139, USA; 7 Department of Surgery, University of Florida College of Medicine, Gainesville, FL 32610, USA; 8 Department of Geriatrics and Gerontology, New Jersey Institute for Successful Aging, Rowan University School of Osteopathic Medicine, Stratford, NJ 08084, USA; 9 Perioperative Cognitive Anesthesia Network (PeCAN), University of Florida, Gainesville, FL 32610, USA

**Keywords:** Clock drawing test, perioperative neuropsychology, transcatheter aortic valve replacement, hospital outcomes

## Abstract

**Aims:**

Reduced pre-operative cognitive functioning in older adults is a risk factor for postoperative complications, but it is unknown if preoperative digitally-acquired clock drawing test (CDT) cognitive screening variables, which allow for more nuanced examination of patient performance, may predict lengthier hospital stay and greater cost of hospital care. This issue is particularly relevant for older adults undergoing transcatheter aortic valve replacement (TAVR), as this surgical procedure is chosen for intermediate-risk older adults needing aortic replacement. This proof of concept research explored if specific latency and graphomotor variables indicative of planning from digitally-acquired command and copy clock drawing would predict post-TAVR duration and cost of hospitalization, over and above age, education, American Society of Anesthesiologists (ASA) physical status classification score, and frailty.

**Methods:**

Form January 2018 to December 2019, 162 out of 190 individuals electing TAVR completed digital clock drawing as part of a hospital wide cognitive screening program. Separate hierarchical regressions were computed for the command and copy conditions of the CDT and assessed how a-priori selected clock drawing metrics (total time to completion, ideal digit placement difference, and hour hand distance from center; included within the same block) incrementally predicted outcome, as measured by R^2^ change significance values.

**Results:**

Above and beyond age, education, ASA physical status classification score, and frailty, only digitally-acquired CDT copy performance explained significant variance for length of hospital stay (9.5%) and cost of care (8.9%).

**Conclusions:**

Digital variables from clock copy condition provided predictive value over common demographic and comorbidity variables. We hypothesize this is due to the sensitivity of the copy condition to executive dysfunction, as has been shown in previous studies for subtypes of cognitive impairment. Individuals undergoing TAVR procedures are often frail and executively compromised due to their cerebrovascular disease. We encourage additional research on the value of digitally-acquired clock drawing within different surgery types. Type of cognitive impairment and the value of digitally-acquired CDT command and copy parameters in other surgeries remain unknown.

## Introduction

Although preoperative cognitive impairments are well known predictors of negative cognitive postoperative outcomes [1–6], it remains unknown if preoperative cognitive screening metrics provide valuable predictive information relevant to hospitals providing the care for older adults electing surgical procedures. One relevant surgical procedure is transcatheter aortic valve replacement (TAVR), the procedure of choice relative to surgical aortic valve replacement in intermediate-risk older adult patients [7]. Although TAVR is associated with reduced mortality relative to standard surgical aortic valve replacement, the procedure can still be accompanied by negative complications impacting hospital cost and stay, e.g., Tilley and colleagues [8] reported that 23% of patients aged 60 years or older who underwent TAVR experience delirium, a preventative cause of cognitive impairment and dementia.

Towards improving hospital care modeling for TAVR, researchers have largely examined the predictive value of traditional metrics such as age and comorbidity on TAVR outcome costs [9], and more recently frailty as a predictor of hospital stay [10–12]. Integrating preoperative cognitive biomarkers into the model is the next logical research approach, particularly given: (1) the abundance of research documenting the significance of preoperative cognition on postoperative outcome [6]; (2) that 54% of all surgical procedures are performed on older adults [13]; (3) prospective hospital-based studies show at least 20% of older adults electing surgical procedures have mild to moderate cognitive limitations on standardized screening metrics [14, 15]; and (4) general surgery guidelines strongly recommend at minimum preoperative cognitive screening for older adults electing surgery.

It is possible that older adults with even subtle cognitive vulnerabilities undergoing TAVR may remain hospitalized longer with an increased cost of care. Cognitive screenings could serve as a functional biomarker to predict which patients may benefit from improved care planning thereby reducing postoperative hospital costs and stay. It is important, however, to identify a screening tool that can be used by medical staff and provide rapid information yet remain sensitive to subtle effects of abnormal aging [14].

One potential tool is the digitally-acquired clock drawing test (CDT), where patients are first asked to draw a clock to a command and then copy a model of a clock [15–19]. The CDT is a classic neuropsychological tool often used as a cognitive screener for dementia [20, 21]. The test requires an individual to “draw the face of a clock, put in all the numbers and set the hands to 10 after 11” (known as the command condition), and then copy a model of a clock with the hands set to 10 after 11 (known as the copy condition). While the command condition assesses multiple cognitive components, the copy condition appears more specific to executive and visuospatial planning deficits [21–23]. Approximately ten years ago, this traditional screener was upgraded using digital technology allowing for more nuanced assessment of subtle cognitive vulnerabilities [17]. Prospective research shows validity with digital clock drawing latency and graphomotor variables [16, 18, 24, 25], although many of the variables extracted from this test are still to be examined relative to hypothetical constructs and neuroimaging variables [26, 27].

The present proof of concept study assessed whether specific digitally-acquired latency and graphomotor CDT variables acquired during a preoperative hospital visit prior to TAVR would explain any variance in length of post-TAVR stay and hospital cost, even after considering patient age, education, comorbidity, and frailty. Typically, length of stay and hospital cost investigations include thousands of data-points [9]. Any explained variance from this preliminary investigation may provide support for larger-scale investigations across hospital networks. Although previous research has demonstrated CDT copy acquired from traditional hand-scoring methods significantly predicted length of stay in older adults undergoing elective surgery [14], the current investigation will be the first to address the question with digital assessment technology. Given that individuals with TAVR have cerebrovascular disease [28] which associates with executive and graphomotor processing speed limitations [29, 30], we a-priori explored the predictive value of CDT total clock drawing time [24] and key graphomotor metrics assessing visual attention and planning [31, 32].

## Materials and methods

This manuscript adheres to the applicable STROBE guidelines.

### Study setting and participants

The present study was conducted at the University of Florida (UF) and UF Health. Data were acquired via a federally-funded investigation and institutional review board-approved protocol with Health Insurance Portability and Accountability Act (HIPAA) waiver and honest broker medical record extraction. The study followed Declaration of Helsinki standards and was in accordance with all institutional guidelines. Inclusion: primary English speaking adults age 65 or older electing TAVR within UF Health from the periods of Jan 2, 2018 to Dec 31, 2019 and triaged for an in-person visit within the UF Preoperative Anesthesia Clinic using the Patient-Centered Anesthesia Triage System criteria as defined by Enneking and colleagues [33] as part of routine medical care. Exclusion: non-English speaking or not fluent in the English language; education < 4 years; visual, hearing, or motor extremity limitation that would preclude a valid clock drawing production.

### Procedure

Licensed neuropsychologists and doctoral-level neuropsychology students trained preoperative anesthesia medical staff, including residents and attending physicians, advanced registered nurse practitioners, and registered nurses on administration of the Preoperative Frailty-Cognitive Protocol with particular attention paid to reliable administration of digitally-acquired clock drawing. As described in Amini et al. [14], training occurred in two segments. Group-based education involved separate team meetings to explain the necessity for reliability and accuracy. Individual trainings involved having a trained instructor administering the protocol to each staff member and then requiring the staff members to administer the protocol to another team member with the instructor present. Each day, a neuropsychology instructor visited each team member to assess protocols for administration problems, train new staff members (rotating residents), and troubleshoot questions.

Each preoperative testing room contained a binder with a packet of materials for reference and administration, as well as a hand dynamometer used to assess for frailty [34]. Packets included instructions for frailty assessment, how to record education, and digitally-acquired CDT administration. The clock drawing to command was administered first, followed by the copy test condition. Each staff member placed a patient identification sticker on the paper containing patient name, medical record number, date of birth, age, sex, appointment encounter number, and admission date. Completed protocols, including the digital clock drawing data, were transferred into the patient’s electronic medical record, with electronic flags communicated ahead of time to the institutional review board honest data broker to enable record retrievals according to institutional review board approval.

### Digitally-acquired CDT

Each participant completed clock drawing to command and copy on “smart paper” using an Anoto pen that records pen positioning 80 times per second with a spatial accuracy of 0.005 cm. As part of the study protocol, test administration was completed with a trained preoperative anesthesia staff member. Patients were presented with 21.59 cm × 27.94 cm smart paper folded in half giving the patient an area of 21.59 cm × 13.97 cm to draw their clock. Data from the digital pen are then downloaded into an in-house software system (CDT classification assist tool [17]) trained to classify each pen stroke with at least 84% accuracy into separate grouping categories. Then, a trained rater with high accuracy (93–99%) reviews the computer software classifications for accuracy before the clock scoring is finalized for data entry. For command condition, participants were instructed to “draw the face of a clock, put in all the numbers, and set the hands to 10 after 11 [21].” After the command condition, the participants were presented with a model clock and asked to copy it (known as the copy condition). The following are latency and graphomotor outcome variables of interest.

#### Latency

Total time to completion: the amount of time (s) from the moment the participant begins the first pen stroke of the clock drawing to the moment the participant finishes the last pen stroke. This measurement was acquired for both the command and copy conditions separately. Previous research demonstrated that on command condition, longer total time to completion associated with worse performance on tests measuring processing speed, language, working memory, and declarative memory, while copy time to completion associated with reduced processing speed and working memory [16].

#### Graphomotor

We examined two experimental graphomotor metrics hypothesized to measure executive abilities including visual planning and visuo-spatial organization [26], and vulnerable to the effects of aging [35].

1. Ideal digit placement difference: the total difference (degrees) in each digit placement on the participant’s clock drawing from the ideal-correct placement is calculated via summation of all 12 digit-differences in degrees from their ideal placement. Individuals who drew less than 12 digits, more than 12 digits, or who did not draw a clockface were excluded from these analyses (command: 20 excluded; copy: 12 excluded). Unpublished (in submission) research from our laboratory assessing 201 older adults age 55 and up who are either cognitively healthy or meet criteria for mild cognitive impairment [36] shows that the digit misplacement variable to command negatively and significantly associates with confrontation naming ability (Boston naming test [37]; *r* = −0.303, *P* < 0.001), reasoning (Wechsler Abbreviated Scale of Intelligence, Matrix Reasoning [38]; *r* = −0.248, *P* < 0.001), and visuospatial attention (Judgement of Line Orientation [39]; *r* = −0.248, *P* = 0.003), such that greater digit misplacement correlated with worse neuropsychological test performance. Digit placement did not associate with processing speed (Stroop word reading [40]; *r* = −0.070, *P* = 0.413), working memory (Wechsler Adult Intelligence Scale-Third Edition, Digit Span Backward [41]; *r* = −0.134, *P* = 0.114), or episodic memory (Wechsler Memory Scale-Third Edition, Logical Memory II [42]; *r* = −0.106, *P* = 0.212).
2. Hour hand distance from center: distance from the inner end of the hour hand to the center of the clock face (millimeters). We extracted this variable based on prior research demonstrating increased upward attentional bias with aging [31, 32]. It has been posited that the age-related reductions in the dorsal attention stream, mediated by the parietal lobe, may be responsible for allocating downward attention [31, 32]. With aging there is degradation of this dorsal stream, inducing an upward attentional bias [31, 32]. Therefore, when drawing a clock, it is possible that older adults with even subtle changes in visuospatial and planning abilities may deviate when determining the center of the clock (the central axis of the hour and minute hands). Unpublished research (from the aforementioned study) found that hour hand distance from center to command negatively and significantly associates with confrontation naming ability (Boston naming test [37]; *r* = −0.152, *P* < 0.05) and processing speed (Stroop word reading [40]; *r* = −0.159, *P* = < 0.05). Hour hand distance from center did not associate with reasoning (Wechsler Abbreviated Scale of Intelligence, Matrix Reasoning [38]; *r* = −0.076, *P* = 0.311), visuospatial attention (Judgment of Line Orientation [39]; *r* = −0.105, *P* = 0.165), working memory (Wechsler Adult Intelligence Scale-Third Edition, Digit Span Backward [41]; *r* = −0.003, *P* = 0.970), or episodic memory (Wechsler Memory Scale-Third Edition, Logical Memory II [42]; *r* = −0.044, *P* = 0.557).

### Other predictor variables of interest

“Age” was defined in years of age at the time of test administration. “Years of formal education” was recorded as the number of years of formal education completed. Skipped years counted toward the total number of years. Repeated years did not add additional years. If the patient dropped out of high school, staff recorded how many full years of school the patient completed. For example, a high school graduate counted as 12 years; a bachelor’s degree counted as 16 years, and so forth. “Comorbidity” was defined by the American Society of Anesthesiologists (ASA) physical status classification [43] with scores ranging from 1 to 5, with 1 representing completely healthy and fit; 2 mild systemic disease; 3 severe systemic disease; 4 incapacitating disease that is a constant threat to life; and 5 being a patient who is not expected to live 24 h with or without surgery. “Frailty” was defined using the criteria from Fried et al. [34]. A patient qualified as frail if he/she exhibited/reported ≥ 3 of the following: (1) unintended weight loss of ≥ 4.54 kg within the last 6 months; (2) subjective exhaustion, defined as endorsing moderate feelings that everything he/she did was an effort over the last week or moderate feelings that he/she could not “get going” in the last week; (3) slow walking speed (4.57 meters in ≥ 7 s for men below 1.73 meters and women below 1.60 meters; 4.57 meters in > 6 s in men above 1.73 meters and women above 1.60 meters); (4) grip strength below a normative cutoff defined by the geriatrics evaluation and management tools [44], and (5) low physical activity as defined by the Duke Activity Status Index [45]. The final outcome variable was scored of 0–5, where 0–1 = no frailty, 2–3 = prefrailty, and 4–5 = frail.

### Hospital outcomes

All hospital outcome variables were acquired from electronic medical records provided through an institutional review board (IRB) sanctioned honest broker. Dependent variables of interest include length of hospital stay (hours) and unadjusted total cost of hospital care (US Dollars; USD). Additional outcomes collected as sample descriptors include discharge placement and 30-day mortality.

### Statistical analyses

Data are available upon reasonable request to the corresponding author.

Data were checked for implausible values, missingness, and distributional form. Digitally-acquired CDT variables were assessed for multicollinearity. Due to non-normality, CDT hour hand distance from center and total time to completion were log transformed; length of stay and cost of care were square root transformed. Statistical significance was set to 0.05 and tests were two-sided.

It is known that age, educational attainment, comorbidities, and frailty serve as risk factors for negative outcomes following surgery. We aimed to understand the contribution of cognitive performance to negative postoperative outcomes, over and above the risk associated with age, education, comorbidities, and frailty. The contribution of cognition to hospital outcomes was assessed via separate hierarchical regressions with length of stay and cost of care as the dependent variables. Age, education, ASA score [43], and Fried frailty [34] score were entered into block one, and block two consisted of CDT total time to completion, ideal digit placement difference, and hour hand distance from center. Separate models were computed for the command and copy conditions of the CDT. R^2^ change values were used to assess whether or not clock drawing performance to either command or copy conditions significantly improved the prediction of length of stay and cost of care, over and above age, education, ASA score [43], and frailty [34].

## Results

### Participants

A total of 190 individuals 65+ underwent TAVR during the period of Jan 2, 2018 to Dec 31, 2019. Of those, 162 met inclusion criteria and completed a preoperative, digitally-acquired CDT to command and copy conditions (age: 78.77 ± 7.04, range 65–97; education years: 13.86 ± 3.11, range 4–26; sex: 58% male; race: 96% Caucasian; ASA score: 3 = 18.5%, 4 = 77.2%, 5 = 0.6%, missing = 3.7%; frailty: 0 = 19.1%, 1 = 14.8%, 2 = 17.3%, 3 = 17.9%, 4 = 18.5%, 5 = 4.3%, missing = 8.0%; length of stay: 62.01 ± 60.23 h; and unadjusted cost of care: $150,931.65 ± 62,070.48 USD). Missing data were identified for education (*n* = 10), ASA (*n* = 6), and Frailty (*n* = 13).

Upon discharge, 132 individuals (81.98%) returned home without additional care, 19 returned home with additional care, 7 entered skilled nursing facilities, 1 entered long-term care, and 3 entered rehabilitation. Two individuals (1.2%) died within 30 days of the procedure.

### CDT performance as predictors of hospital outcomes

Separate models were computed for the command and copy conditions of the CDT. Command condition: average time to completion was 45.42 ± 28.72 s, average ideal digit placement difference was 134.55 ± 138.17 degrees, and average hour hand distance from center was 4.17 ± 3.27 mm. Copy condition: average time to completion was 32.92 ± 15.17 s, average ideal digit placement difference was 105.66 ± 160.65 degrees, and average hour hand distance from center was 3.57 ± 2.44 mm.

**Figure 1.**
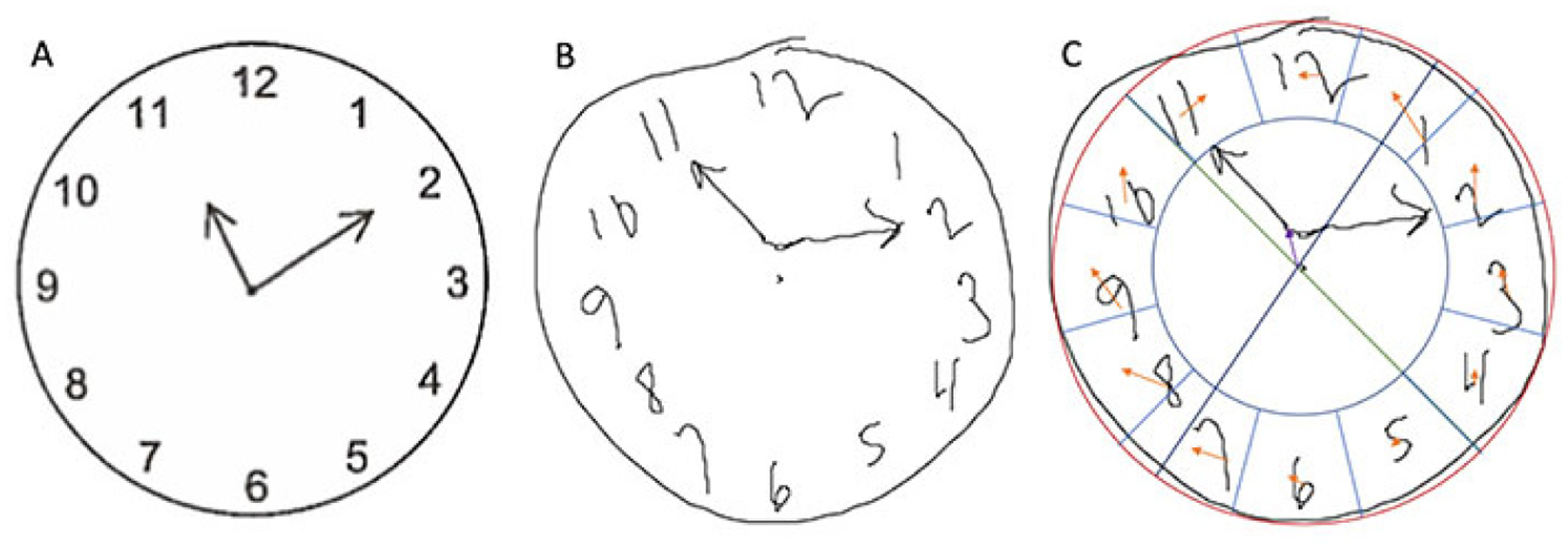
Participant copy condition clock drawing demonstrating hour hand distance from center and ideal digit placement difference. (A) Model clock presented to participants in the CDT copy condition; (B) drawing of a copy condition clock by a participant with longer-than-average length of stay (103.73 h) and cost of care ($ 211,159.88); (C) representation of hour hand distance from center and ideal digit placement difference for the participant’s clock. Red: circle of best fit; Green and dark blue: axes to identify center of circle; Purple: hour hand distance from center; Light blue: region for placement of each digit; Orange: distance of each digit from the ideal placement

1. Length of Stay: block one: age, education, ASA score, and frailty significantly explained 10.2% of the variance in hospital length of stay (R^2^ = 0.102, *P* = 0.015). Block two: CDT copy performance measures significantly increased R^2^ by 0.095, explaining an additional 9.5% of variance in length of hospital stay (R^2^ change = 0.095, *P* = 0.006); (Table 1, Figure 1).
2. Cost of Care: block one: age, education, ASA, and frailty did not explain a statistically significant amount of variance in cost of care (R^2^ = 0.045, *P* = 0.251). Block two: CDT copy test parameters increased R^2^ by 0.089, indicating an additional 8.9% of variance explained in cost of care (R^2^ change = 0.089, *P* = 0.012); (Table 1, Figure 1).

CDT performance on the command condition did not significantly explain variance in either model (both *P* > 0.05).

## Discussion

The copy condition of the digitally-acquired CDT may be more sensitive than the command condition to subtle, preoperative cognitive impairments capable of predicting hospital outcomes and resource use after TAVR for adults older than 65. This expands upon previous research assessing the predictive value of manually scored clock drawing to command and copy in a preoperative anesthesia screening facility [14]. The present study demonstrates that specific outcome measures of copy clock drawing, but not command clock drawing, predict variance in length of hospital stay and cost of care above-beyond age, education, ASA score, and frailty in a TAVR population. Specifically, copy total time to completion was a significant predictor of both length of stay and cost of care, while hour hand distance from center was a predictor of length of stay.

Individuals who produce errors on the copy condition, and consequently take longer to complete the copy test condition, have been shown to have more executive deficits [21–23]. Previous research has shown that individuals with primary executive deficiencies and visuospatial deficits will produce similar errors on the copy condition relative to the original command condition (i.e. individuals with subcortical diseases such as small vessel vascular disease, Parkinson’s disease, or Huntington’s disease) [23, 46]. Hypothetically, individuals with subcortical diseases and executive dysfunction may be more vulnerable to perioperative stressors, as research suggests older adults with such executive difficulties prior to and following surgery have greater difficulties with post-discharge instrumental activities of daily living coupled with greater caregiver burden [1]. Taken together, the present findings support previous arguments demonstrating the efficacy of including both CDT conditions [22], but in preoperative settings, and particularly for individuals where vascular and/or executive impairment may be most prevalent. Although the current study demonstrated value for the copy condition in predicting hospital outcomes, future research investigations with larger and more diverse surgery types are warranted.

Within the clock copy condition the measurement of total time to completion and hour hand distance from center were the leading variables significantly adding to the overall statistical models for total length of stay and hospital cost. Total time to completion has been shown to increase with age and involves both a graphomotor component and non-motor cognitive abilities [24]. Previous research has also demonstrated that copy total time to completion primarily associates with processing speed and working memory [16], which are two cognitive functions that tend to decline with age [47] and are highly vulnerable to cerebrovascular changes [48].

At this time, it is unclear why CDT variables of interest in the command condition did not predict hospital outcomes. Total clock drawing time to command, for example, associates with numerous cognitive domains [16]. Given the many cognitive domains involved in drawing a clock to command, it may be that the command condition can identify impairment in any cognitive domain, while individuals who show errors on copy demonstrate executive “risk” necessitating closer monitoring for postoperative care. This may be particularly true for intermediate-risk surgical individuals such as those electing TAVR. Further research assessing command time to completion, hour hand distance from center, and ideal digit placement difference across larger and diverse patient groups is warranted.

The graphomotor variables of hour hand distance from center and ideal digit placement difference are, theoretically, more sensitive to neurodegenerative changes within the dorsal attentional stream, as with aging there is atrophy of the dorsal stream which may lead to an upward attentional bias [31, 32]. Of these two variables, hour hand distance from center showed potential value for predicting length of stay. Again, it was only when these hands were misplaced in the copy condition that significant variance was explained. Collectively, these results provide rationale for larger research investigations while also appreciating the cognitive changes and type of difficulties individuals electing TAVR experience postoperatively.

We recognize study limitations. These findings are preliminary due to our limited sample size and because two of the cognitive variables assessed (hour hand distance from center and ideal digit placement difference) are exploratory in nature. Additionally, we identified missing data in the variables of education, ASA, and frailty. Retrospective review of the data indicated that nursing staff did not report these variables and reasons for this are unknown. Moreover, we have limited details regarding the type of complications encountered in these patients, or reasons for the increased length of stay and cost (e.g., delirium). Our sample was very homogeneous, as the majority were Caucasian. However, TAVR patients nationwide are most often Caucasian men and of a mean age of 80 years old [49]. We also recognize that while our findings speak to the utility of a digitally-acquired version of the CDT, we do not compare the digitally-acquired CDT to other cognitive measures, either with or without a digital adaptation.

Despite these limitations, the present findings highlight the promise of digitally-acquired neuropsychological tools for detecting subtle cognitive vulnerabilities that may predict negative postoperative outcomes. This is the first published study to assess a digitally-acquired cognitive assessment in well-characterized TAVR patients specifically. Study strengths include the digital technology integration, theoretical approach to clock drawing constructs, and the addition of a copy condition relative to many assessments which solely employ command clock drawing [22, 50]. In addition, these proof of concept findings lend evidence for the utility of similar investigations in larger and more diverse patient samples. The findings in the present proof of concept paper are novel and underscore the value of digitally-acquired neuropsychological parameters that cannot be efficiently measured using traditional paper and pencil tests. Future research is needed to investigate additional digitally-acquired CDT variables and whether or not they, too, have predictive value for hospital outcomes following surgery.

## Figures and Tables

**Table 1.** Results from hierarchical regressions demonstrating cognitive performance via individual command and copy digitally-acquired clock drawing test predictors of length of stay and cost of care

Outcome variable	Clock variable	Standardized beta	*t*	Sig.
Length of stay	Command			
	Total time to completion (seconds)	0.24	2.6	0.011
	Ideal digit placement difference (degrees)	0.12	−0.13	0.901
	Hour hand distance from center (millimeters)	−0.03	−0.27	0.787
	Copy			
	Total time to completion (seconds)	0.23	2.49	0.014
	Ideal digit placement difference (degrees)	0.17	1.93	0.057
	Hour hand distance from center (millimeters)	0.22	2.57	0.011
Cost of care	Command			
	Time to completion (seconds)	0.18	1.94	0.055
	Ideal digit placement difference (degrees)	−0.06	−0.6	0.548
	Hour hand distance from center (millimeters)	−0.02	0.19	0.852
	Copy			
	Time to completion (seconds)	0.27	2.78	0.006
	Ideal digit placement difference (degrees)	0.16	1.72	0.088
	Hour hand distance from center (millimeters)	0.18	1.95	0.054

*t*: *t*-value; Sig.: significance (*P*-value)

## Abbreviations

ASA: American Society of Anesthesiologists
CDT: clock drawing test
TAVR: transcatheter aortic valve replacement
